# Recent Advances in Particle Design for High-Concentration Protein Suspension Injectables

**DOI:** 10.3390/pharmaceutics18040450

**Published:** 2026-04-07

**Authors:** Yijing Huang, Chanakya D. Patil, Kinnari Santosh Arte, Jiaying Liu, Haichen Nie, Qi Tony Zhou, Li Lily Qu

**Affiliations:** 1 Department of Molecular and Industrial Pharmaceutics, College of Pharmacy, Purdue University, West Lafayette, IN 47907, USA; huan1611@purdue.edu (Y.H.); patilc@purdue.edu (C.D.P.);; 2 Sterile Product Development, Pharmaceutical Sciences & Global Clinical Supply, Merck & Co., Inc., Rahway, NJ 07065, USA

**Keywords:** high-concentration proteins, subcutaneous injectables, suspension, particle production, particle properties, viscosity, injectability, sedimentation

## Abstract

Subcutaneous administration has become an increasingly important route for delivering protein therapeutics, driven by patient convenience and the growing use of self-administration devices. However, conventional subcutaneous injection systems are typically limited to injection volumes of approximately 1–2 mL, posing significant formulation challenges for protein drugs requiring high therapeutic doses. Monoclonal antibodies (mAbs), for example, often require concentrations exceeding 100 mg/mL to enable subcutaneous delivery, which introduces challenges related to limited solubility, elevated viscosity, and an increased risk of physical and chemical instability. Therefore, high-concentration protein suspensions have emerged as a promising formulation strategy to overcome these limitations and enable subcutaneous administration of high-dose proteins. In such systems, therapeutic protein solid particles are suspended in vehicles in which they are insoluble, giving rise to unique considerations related to particle properties, protein stability, and suspension behaviors such as viscosity, injectability, and sedimentation. Accordingly, multiple particle production approaches have been explored to enable the development of ultra-high-concentration protein suspensions (>200 mg/mL). This review article aims to provide a comprehensive overview of particle formation techniques and the relationships between key particle properties and suspension performance attributes relevant to the development of high-concentration protein suspensions for injectable applications, as well as future directions in this field.

## 1. Introduction

The global market for therapeutic proteins, including recombinant proteins, enzymes, cytokines, and monoclonal antibodies (mAbs), was valued at approximately $168.5 billion in 2020 and is expected to continue growing [1,2]. In 2024, a total of 13 mAbs were approved by the United States Food and Drug Administration (FDA), which was the most approved drug class [3]. MAbs primarily target cancer and immunological diseases, which often require high doses for efficacy [4,5]. More than half of the mAb products have a dose of 200 mg or beyond per administration [4]. MAbs are typically formulated in aqueous buffers for intravenous or subcutaneous injections. Compared to intravenous administration, subcutaneous administration reduces healthcare burden and supports patient-centric treatment strategies by enabling self-administration products [1,6]. However, conventional subcutaneous devices typically have an injection-volume limit of approximately 2 mL. Therefore, high-concentration mAb formulations (>100 mg/mL) are necessary for the development of subcutaneous mAb products.

High-concentration mAbs can lead to protein instability and high viscosity in aqueous solutions, posing significant challenges for the development of subcutaneous drug products [7]. Current strategies to mitigate instability, such as protein aggregation at high concentrations, include adding surfactants such as polysorbates and poloxamers, and sugars like trehalose and sucrose, as well as the application of lyophilization, which can efficiently enhance storage stability [6,8]. However, the use of surfactants, particularly widely used polysorbates, has become an increasing concern due to their potential degradation over time, which can negatively affect protein stability [9,10]. While lyophilized products represent approximately 6% of the marketed high-concentration protein drugs, they often require extended reconstitution times at high concentrations, which may preclude the possibility of self-administration using devices such as prefilled syringes or autoinjectors [6,11,12,13].

High viscosity complicates manufacturing processes (e.g., filtration and filling) and limits the injectability of high-concentration protein drugs. Studies have shown that the viscosity of mAb solutions can rise exponentially once it reaches a certain concentration threshold [14]. Efforts have been made to utilize viscosity-reducing agents (VRAs) to reduce viscosity, including arginine, sodium chloride, proline, glycine, and lysine [15]. However, due to the limited fundamental understanding of rheological behaviors of various mAb solutions, empirical excipient screening for VRAs remains the primary approach, which is often time-consuming and difficult to apply to other products [16]. In parallel, co-formulation with hyaluronidase has been used in a few drug products to transiently increase the subcutaneous injection volume beyond 2 mL [1,17]. This is achieved by degrading hyaluronan, a key component of the subcutaneous extracellular matrix that creates resistance to bulk fluid flow and limits the injectable volume for subcutaneous administration [16,18]. For example, subcutaneous Keytruda (pembrolizumab) co-formulated with hyaluronidase was approved by FDA in 2025 [19]. This product enables pembrolizumab concentration to be as high as 165 mg/mL with an injection volume up to 4.8 mL [19]. However, the addition of hyaluronidase may raise concerns regarding its compatibility with certain drugs, potentially introducing instability issues, analytical challenges, and even risks of tissue damage [1,13].

Wearable devices or on-body delivery systems (OBDS) also enable the subcutaneous administration of larger volumes (approximately 3–25 mL) [1,20]. For example, EMPAVELI^®^ (pegcetacoplan), a peptide therapeutic, is formulated for abdominal subcutaneous delivery via an OBDS at a volume of 20 mL [21]. Ultomiris^®^ (ravulizumab) is formulated at a protein concentration of 70 mg/mL with a total delivery volume of 3.5 mL and is administered via an OBDS equipped with a prefilled cartridge [22]. However, these drug–device combinational products complicate manufacturing, prolong development timelines, increase costs and prices, and face more complex regulatory requirements [1,20].

In addition to these strategies, the development of low-volume, high-concentration protein suspensions has emerged as a promising platform to mitigate protein instability and viscosity limitations. This concept was first reported by Yang et al. in 2003, who demonstrated that therapeutic mAbs could be crystallized in batches and formulated as crystalline mAb suspensions [23,24]. These suspensions exhibited substantially lower viscosity at 200 mg/mL than the corresponding solutions, while maintaining protein stability [23,24].

Beyond crystallization, other solid protein production approaches, such as milling of protein lyophilizates and spray drying of protein formulations, have subsequently been employed to develop high-concentration protein suspensions. In these formulations, protein particles are dispersed in vehicles in which particles exhibit low solubility, using mixing techniques, including stirring, shaking, vortexing, and homogenization, to form low-volume, high-concentration protein suspensions [25,26,27,28].

These protein suspensions have been shown to reduce viscosity, as discussed later in Section 2. In protein solutions, viscosity arises from intermolecular interactions among protein molecules, including hydrodynamic, electrostatic, hydrophobic, and van der Waals interactions, which can promote the formation of transient protein networks that resist flow [15,29,30]. As a result, the viscosity of protein solutions can increase substantially at high protein concentrations. In contrast, in protein suspensions, the formation of protein particles can enable dense packing of the protein and reduce its effective excluded volume, while also hindering the formation of extended protein networks in the continuous phase, thereby potentially lowering viscosity relative to protein solutions [31]. However, these concentrated protein suspensions can also exhibit non-Newtonian behavior, such as shear thinning, as discussed in Section 2 and Section 3. This suggests that the presence of concentrated particles introduces additional complexity to the interaction network within the suspension. In general, suspension viscosity is governed by both the viscosity of the vehicle and the particle–particle, particle–vehicle, and vehicle–vehicle interactions [26]. Therefore, the general statement that “a suspension exhibits lower viscosity than the corresponding solution” may not always hold true unless the relevant conditions, including concentration and shear rate, are clearly specified.

Moreover, several companies pursuing this formulation strategy, including Xeris [32], Elektrofi [33], Lindy Biosciences [34], and Nanoform [35], have partnered with big pharma companies to develop high-concentration suspension injectables for various biologics. These activities collectively demonstrate the strong potential and growing interest in this formulation technique.

Overall, protein suspensions represent an emerging formulation strategy to develop low-volume, high-dose pharmaceutical injectables. Given the recent progress in this area and the limited number of review articles focusing on particle design and the interplay between particle properties and suspension performance, this review comprehensively summarizes and discusses different particle production methods with a particular emphasis on particle design and particle properties. We also examine how different particle properties, including particle size distribution, particle morphology, and particle density, influence overall suspension performance, particularly viscosity, injectability, and sedimentation. Understanding the interplay between particle properties and suspension performance can provide meaningful guidance for particle design. Although the FDA has not yet defined “high-concentration” for protein drug products, in this review, “high-concentration” refers broadly to protein suspensions formulated at protein concentrations above 100 mg/mL, and “ultra-high-concentration” refers to those above 200 mg/mL.

## 2. Particle Formation Techniques for High-Concentration Protein Suspensions

This section discusses these different approaches of particle formation for developing protein suspensions with a particular focus on recent advances.

### 2.1. Protein Precipitation

Crystalline forms of therapeutic proteins offer several advantages, including facilitating protein isolation and purification during manufacturing, enabling efficient protein concentration for high-dose therapeutics, and providing improved stability relative to amorphous proteins [36,37]. Similar to small-molecule compounds, protein crystallization involves nucleation and crystal growth [38]. Protein crystallization typically occurs from a supersaturated protein solution upon the addition of a precipitant, such as a salt or a polymer [37]. Crystalline protein suspensions have been reported to develop low-viscosity, high-concentration formulations for subcutaneous delivery [39]. For example, crystalline infliximab suspended in a buffer containing polyethylene glycol (PEG) and ethanol demonstrated a viscosity below 50 centipoise (cP; 1 cP = 1 mPa*s) and acceptable injectability at 200 mg/mL, whereas the infliximab solution exhibited a viscosity of 275 cP at 150 mg/mL [23,24]. It should be noted that viscosity was measured using a Cannon–Fenske viscometer, a U-shaped capillary instrument designed to determine the kinematic viscosity of transparent or opaque Newtonian liquids. No other rheological characterization was reported for these crystalline mAb suspensions, despite their favorable injectability [23]. Moreover, the authors demonstrated that the suspension formulation did not affect the in vitro biological activity of the protein [23]. In addition, the crystalline mAb suspension did not induce inflammatory reactions at the subcutaneous injection site in mice, exhibited a pharmacokinetic profile comparable to that of the corresponding solution formulation after subcutaneous injection in rats, and demonstrated dose-dependent efficacy in mice [23].

Protein crystallization, especially for the full-length mAbs, remains substantially challenging due to their large size, surface oligosaccharides, and high segmental flexibility [23]. Consequently, only a few studies have investigated high-concentration crystalline mAb suspensions with low viscosity [39,40,41,42]. For example, Reichert et al. investigated the effect of microgravity on the mAb crystallization process to better understand crystal growth behavior [40]. As a result, uniform mAb crystals were generated at the 1 mL scale using vertical rotation, yielding crystalline suspensions in (4-(2-hydroxyethyl)-1-piperazineethanesulfonic acid) (HEPES) buffer containing PEG [40]. This approach improved rheological properties of the crystalline mAb suspensions [40]. The resulting crystalline mAb suspensions could be further concentrated by centrifugation to approximately 140 mg/mL [40]. However, in this study, all characterizations were performed after dilution to protein concentrations below 100 mg/mL [40]. Evaluation of such crystalline suspensions at higher concentrations would be more informative. Additionally, this method requires further validation in terms of scalability and adaptability.

In addition to the crystalline protein suspension, Srinivasan et al. illustrated the precipitation of amorphous bovine γ-globulin via cold absolute ethanol in the solution, followed by filtration and multiple drying steps to obtain the dried globulin [43]. The resulting material was characterized as an ultrafine, white, and free-flowing powder [43]. It was further suspended in various non-aqueous vehicles under magnetic stirring, followed by centrifugation to produce the amorphous globulin suspensions at desired concentrations. All the globulin suspensions demonstrated lower viscosity than the corresponding aqueous solution at 260 mg/mL, where the lowest one was only 3.6 cP for the suspension in tetrahydrofuran [43]. However, there are safety concerns associated with using these neat non-aqueous vehicles in injectables [44,45]. Furthermore, this study evaluated the roles of hydrophobic interactions, electrostatic interactions, and hydrogen bonding in governing suspension viscosity [43]. The results highlighted the importance of hydrogen bonding between non-aqueous vehicles and protein particles, showing that suspending vehicles containing zero or one hydrogen bond donors produced the lowest globulin suspension viscosities [43].

Moreover, several studies summarized in Table 1 have encapsulated such crystalline or amorphous proteins into hydrogel particles to prepare protein suspensions in aqueous vehicles, achieving protein concentrations of up to 300 mg/mL [41,42,46]. Hydrogel particles made of crosslinked hydrophilic polymers are typically soft and lubricious, and exhibit shear-thinning rheological properties. Hence, hydrogel particles are readily deformable and can be packed to high volume fractions before jamming occurs, making them suitable for high-dose therapeutic delivery [41,42,46]. Regarding the hydrogel encapsulation procedure, centrifugal extrusion induced less protein aggregation than the microfluidic mixing (Table 1) [42]. However, although the overall procedure did not negatively affect protein stability or binding activity and hydrogel encapsulation did not alter in vivo absorption (Table 1), the additional encapsulation step into hydrogel particles complicates the overall particle production process and can introduce increased variability in encapsulation efficiency. This approach also relies on centrifugation to achieve high concentrations, which limits scalability and constrains the maximum achievable protein concentrations.

Overall, protein precipitation could be inherently less predictable and difficult to scale into a robust and reproducible process for protein particle production. Hydrogel encapsulation further complicates process control. Although crystalline proteins offer several attractive advantages, a fundamental mechanistic understanding of the protein crystallization process is required to enhance process reliability and expand clinical applicability.

### 2.2. Milling of Protein Lyophilizates

Lyophilization, or freeze-drying, has been the primary and most widely used technique for producing protein solid formulations [47]. Protein solution formulations are typically filled into glass vials, after which water is first frozen and subsequently removed by sublimation from the vials under reduced pressure [48]. As a result, lyophilized protein formulations form solid cakes in vials, which require a subsequent milling step to generate protein particles suitable for uniform suspension formulations [25,28,43,49].

A few veterinary medications have been formulated by suspending milled lyophilizates of growth hormones in non-aqueous vehicles such as sesame oil and Miglyol 812; but these suspensions are of high viscosity and thereby require large needles (14-gauge to 16-gauge) to be injectable [49,50,51]. For humans, however, the desirable needle sizes for subcutaneous injection are between 25-gauge (25 G) to 27 G [52]. In 2009, Miller et al. developed a high-concentration lysozyme suspension by milling the protein lyophilizates [49]. The lyophilized lysozyme was first milled with a mortar and pestle for several minutes, then sieved through a 400-mesh screen to collect lysozyme particles smaller than 37 µm [49]. The sieved milled lysozyme particles, with an average particle size of 20 µm, were suspended in benzyl benzoate via manual shaking [49]. The resulting suspension exhibited an apparent viscosity of below 30 cP at the lysozyme concentration of 400 mg/mL, which was much lower than the theoretical viscosity (60 cP and increasing sharply at 300 mg/mL; undefined at 400 mg/mL) of the lysozyme solution [49]. In addition, it took approximately 40 s to draw the 1 mL suspension into the syringe via a 25 G needle, demonstrating acceptable syringeability [49]. It should be noted that the viscosity characterized in this study was based on the linear correlation between the time to draw 1 mL of sample and the viscosity, not directly by a rheometer or viscometer [49].

Given that the milling step can produce local heat that may interfere with the stability of thermal-labile proteins, Srinivasan et al. applied liquid nitrogen to create a low-temperature environment to mill the lyophilized murine mAb particles with a mortar and pestle [43]. The murine mAb particles were later suspended in toluene via gentle shaking [43]. This approach successfully prepared a mAb suspension containing 200 mg/mL protein, exhibiting shear-thinning rheological characteristic [43]. The apparent viscosity of the suspension was only 5 cP, whereas the reconstituted solution was 14 cP [43]. The apparent viscosity was determined by linear extrapolation to the zero-shear limit using viscosity values measured at the three highest shear rates [43]. In these two studies [43,49], although the suspension viscosity was lower than that of the corresponding solution, particle production relied on mortar-and-pestle milling, which is neither well controlled nor readily scalable, and is therefore unsuitable for reproducible large-scale manufacture.

In 2021, Marschall et al. optimized this particle production technique by applying standardized milling processes to develop a high-concentration suspension of immunoglobulin G1 (IgG1), a model mAb [25]. Given that milling can introduce substantial mechanical stress and/or generate local heat [53,54], this study first compared the impact of three milling methods on protein integrity: wet media milling, dry milling, and cryogenic milling (cryomilling) [25]. Cryomilling resulted in less protein damage compared with the other two methods, likely due to the low processing temperature [25]. They further chose cryogenic ball milling for the following studies on suspensions [25]. Upon the cryomilling, the lyophilized IgG1 particles showed D_50_ of 7 to 10 µm and D_95_ of 15 to 20 µm with a flake-like morphology [25]. An additional sieving step with a 40 µm mesh was found necessary to ensure the injectability via 25 G needles [25]. The sieved powder was further suspended in various non-aqueous vehicles using an ultrasound bath for 20 min and subsequent manual shaking [25]. The resulting suspension prepared in perfluorohexyloctane (F6H8) at 150 mg/mL protein concentration exhibited a viscosity of approximately 10 cP at a shear rate of 5000 s^−1^ [25].

Notably, the combination of lyophilization, cryomilling, and sieving makes the overall preparation more complex [25]. Lyophilization is time- and energy-consuming, while cryogenic ball milling requires optimization of milling parameters such as the frequency and ball diameter to ensure protein integrity [25]. Moreover, another concern is particle morphology. For these high-concentration suspensions intended for subcutaneous injections, spherical or near-spherical particles are found to minimize needle clogging and improve injectability [28]. However, milled lyophilized particles end up being flake-like and non-spherical, reportedly causing them to fail the injectability test [28,55]. For example, Marschall et al. reported in 2023 that, using the same approach described in their earlier study to produce sieved, milled lyophilizates of lysozyme/trehalose particles, the resulting suspension could not be injected through a 26 G needle while presenting a low viscosity (Table 2) [25,28]. In addition to the difference in suspension protein concentration (150 mg/mL vs. 210 mg/mL; Table 2), this failure was likely due to a particle bridging effect, where milled particles stacked and formed agglomerates at the needle–syringe interface during injection, ultimately impeding the flow [28]. Furthermore, this observation also suggests the poor adaptability of this method across different proteins and mAbs.

Although this technique has demonstrated process stability with respect to protein aggregation [25,28], further efforts are needed to comprehensively evaluate post-storage protein stability, protein binding activity, and in vivo performance, including bioavailability and efficacy, in order to strengthen its potential for clinical translation.

### 2.3. Spray Drying

Spray drying is a one-step, bottom-up drying technique that generates particles directly from a liquid feed using a hot gas stream [56,57]. It is also suitable for continuous manufacturing and adaptable for aseptic operation [57]. Although spray drying is often considered a harsher drying method than lyophilization due to thermal, shear, and air–liquid interfacial stresses, the extremely short drying time substantially reduces the overall stress imposed on proteins. Therefore, with proper stabilizing excipients and process parameters, spray drying has been widely demonstrated to be a viable technique for producing stable biologic solids, including bovine serum albumin (BSA), mAbs, and RNA lipid nanoparticles [57,58,59,60]. Supported by these findings, spray drying is currently the most widely studied approach for developing high-concentration protein suspensions. Table 3 summarizes recent publications on such spray-dried protein suspensions. In these studies, spray-dried protein particles were suspended in non-aqueous solvents to prepare high-concentration suspensions. Most of these formulations have been shown to reduce viscosity, maintain protein stability, and exhibit good injectability (Table 3). It should be noted that injectability results across different studies should be compared with caution and only after alignment of needle gauge, flow rate, device type, and the metric used (e.g., injection force vs. glide force).

In spray-dried protein formulations, trehalose is the most commonly used non-reducing sugar excipient, owing to its high glass transition temperature of 106 °C [61]. An adequate amount of trehalose helps ensure process and storage stability for proteins, but it compromises the suspension’s maximum drug loading capacity. Assuming a protein–stabilizer system is formulated at a 1:1 weight ratio, a 200 mg/mL protein suspension would require a total solid concentration of 400 mg/mL. In addition, a higher total solid concentration usually results in higher viscosity. Hence, reducing the overall excipient amount can increase the particle drug loading and decrease viscosity, aligning with the goal of achieving high-dose, low-viscosity, and injectable suspensions.

**Table 3:** Publications on high-concentration spray-dried protein suspensions.

	Spray-Dried Protein Formulation	Particle Properties	Suspension Vehicle and Suspending Method	Protein Concentration in the Suspension	Suspension Properties Such as Viscosity, Injectability, and Sedimentation **	Reference
1	Three different mAbs; mAb was formulated with trehalose at 2:1 weight ratio.	D_50_ was between 8 µm and 11 µm; morphology was collapsed spherical particles.	Powders were mixed with Miglyol^®^ 840, benzyl benzoate, and ethyl lactate, respectively, by a homogenizer equipped with a 0.5-cm tip for 2 min at 7500 rpm.	Up to 333 mg/mL in ethyl lactate (suspension A)	Suspension A showed a viscosity of below 20 cP and a glide force below 15 N via a 27 G needle at a flow rate of 190 mm/min (1 mL syringe) or approximately 3 mL/min.	[26]
2	An immunoglobulin G2 (IgG2)-type antibody BM1 was formulated with trehalose at various weight ratios-8:2, 6:4, and 4:6.	Average diameter was 2 µm to 8 µm; morphology was collapsed spherical particles.	BM1-trehalose (8:2) was suspended with benzyl benzoate with manual shaking or vortexing (the suspending procedure was not clearly stated).	200 mg/mL	The suspension was shear-thinning. It showed a viscosity of 24 cP at a shear rate of 4000 s^−1^, whereas the reconstituted solution after spray drying had a viscosity of 79 cP. No apparent sedimentation was observed after 5 days of storage at room temperature (RT).	[27]
3	A mAb was formulated with trehalose at a 7:3 weight ratio.	D_50_ was 5.9 µm.	Powders were mixed with different vehicles, respectively, in a cooled ultrasound bath.	Up to 280 mg/mL protein concentration	At 280 mg/mL protein concentration, lowest viscosity (below 10 cP at a shear rate of 5000 s^−1^) and lowest injection force (7.2 N; at 6 mL/min; 27 G needles) were achieved using perfluorobutylpentan (F4H5) as the vehicle, while the reconstituted mAb solution showed a viscosity above 50 cP and a glide force of 38.7 N at 6 mL/min via a 27 G needle.	[28]
	Lysozyme was formulated with trehalose at a 7:3 weight ratio.	D_50_ was 4.5 µm.			At 280 mg/mL, the reconstituted lysozyme solution showed lower viscosity (5.1 cP at a shear rate of 5000 s^−1^) and lower glide force (5 N via a 27 G needle at 6 mL/min) than any lysozyme suspensions prepared in F4H5, F6H8, or perfluorodecalin ***. The F4H5 suspension gave the lowest viscosity and glide force among the suspensions. The F6H8 suspensions at 280 mg/mL and 210 mg/mL did not show sedimentation after 1-month storage at RT.	
4	Bovine serum albumin (BSA), human IgG (hIgG), and an anti-COVID mAb were each formulated with a surfactant copolymer poly(acryloylmorpholine-co-N-isopropylacrylamide) (MoNi) at 20:1 weight ratio.	BSA-MoNi particles were collapsed spherical particles with average diameter of 5 µm to 10 µm.	The MoNi particles formulated with BSA, hIgG, and mAb were each mixed with triacetin by vortexing.	520 mg/mL	The suspension was shear-thinning with viscosity of around 1000 cP at 10 s^−1^ shear rate. The glide force was 14 N at 1 mL/min via a 27 G needle. Minimal sedimentation was seen upon 35-day storage at RT.	[62]
		hIgG-MoNi particles were collapsed spherical particles with average particle diameter of 5 µm to 20 µm.		450 mg/mL	The suspension showed a glide force of 17 N at 1 mL/min via a 26 G needle, and minimal sedimentation upon 35-day storage at RT.	
		mAb-MoNi particles were smooth spherical particles with average particle diameter of 14 µm.		400 mg/mL	The injection force was 6.0 ± 0.1 N through a 26 G needle at 1 mL/min.	
5 *	hIgG solution of 50 mg/mL protein concentration was spray dried. The solution was formulated in glycine buffer with no other excipient information stated.	D_50_ was 8 µm. Morphology was not reported.	Particles were mixed with pre-saturated PEG glycine solution using vortexing for 5 min and subsequent stirring overnight at 400 rpm, leading to the formation of a spray-dried IgG colloidal system. The formulation was placed at RT for a day to remove frothing on the top.	Up to 400 mg/mL	The system was shear-thickening. At 400 mg/mL protein concentration, the viscosity was nearly 800 cP at 1000 s^−1^ shear rate, but the maximum injection force was 16.5 N at a flow rate of 3 mL/min via a 24 G needle.	[63]

* A colloidal system was generated instead of suspending particles directly in the vehicle that cannot dissolve proteins. ** Cross-study comparisons for injectability are limited unless needle gauge, flow rate, device type, and metric definition (injection force vs. glide force) are aligned. *** The viscosity of lysozyme–trehalose (70:30; *w*/*w*) solution did not yet increase drastically at 280 mg/mL protein concentration. For the mAb-trehalose solution, the viscosity increased drastically after 200 mg/mL.

Huang et al. examined the impact of trehalose amount on a spray-dried antibody suspension at various protein–excipient weight ratios (8:2, 6:4, 4:6, and 2:8) [27]. The particles produced at the 2:8 ratio were sticky and difficult to recover from the spray dryer due to the high sugar content [27]. Particles generated at the 8:2 ratio exhibited comparable stability to those of the 6:4 and 4:6 formulations [27]. Hence, the 8:2 particles were selected for the following suspension studies, including viscosity, sedimentation, and protein stability [27]. The resulting suspension was three times less viscous than the corresponding solution, while maintaining protein physical stability [27]. However, identifying the minimum amount of such stabilizers remains largely an empirical process due to the inherent complexity of proteins. To date, among all the reported spray-dried protein suspension publications, the lowest trehalose to protein weight ratio is approximately 1:4 (Study 2, Table 3). 

In parallel, the design of novel excipients providing better protection at lower amounts for spray-dried proteins has recently been reported as a strategy to achieve an ultra-high-concentration suspension formulation of 400–520 mg/mL [62]. In this study, a copolymer, poly(acryloylmorpholine-co-N-isopropylacrylamide) (MoNi), which functions as both a surfactant and a thermal stabilizer during spray drying, was used to produce a stable and injectable BSA suspension at 520 mg/mL with only 5% (*w*/*w*) MoNi (Table 3) [62]. However, the use of novel excipients introduces additional regulatory considerations, which may extend drug development timelines. Moreover, they further evaluated the pharmacokinetic and efficacy profiles of BSA, hIgG, and an anti-COVID mAb in mice, demonstrating that spray-dried protein suspensions did not alter these profiles compared with the corresponding solution formulations [62].

Interestingly, Yadav et al. developed a high-concentration IgG colloidal system by suspending spray-dried IgG in a pre-saturated PEG buffer, resulting in a shear-thickening colloidal system with protein concentrations up to 400 mg/mL [63]. While presenting a high viscosity of nearly 800 cP at a shear rate of 1000 s^−1^, its injection force was only 17 N at a flow rate of 3 mL/min via a 24 G needle [63]. Normally, higher viscosity leads to a larger injection force and poorer injectability [64]. A shear-thickening liquid would exhibit even higher viscosity at elevated shear rates. During the subcutaneous administration, for example, a 10-s injection of 1 mL through a 27 G needle presents shear rates up to 10^5^ s^−1^ [65]. Therefore, the injection force via a 24 G needle was assumed to be much higher than 17 N [63]. This viscosity-injectability paradox might be attributed to (1) the excipients added in the system that could function as molecular lubricants; (2) the syringe-needle device used in that study that could help with the injection [63]. However, further investigation is required to confirm whether the excipient addition or the injection system is a key contributor to the reduced injection forces and to establish a clearer understanding of such a paradox. It should be noted that reports of low injection force vs. shear-thickening system are likely protocol/device-dependent and should not be generalized without standardized testing.

Xeris, one of the companies developing its proprietary platform, XeriJect^®^, also applies spray drying to engineer protein particles and further blends the particles with non-aqueous solvents using a planetary-orbital mixer to prepare high-concentration protein suspensions [66]. The suspensions were reported to be a high-viscosity, shear-thinning, and paste-like semisolid [66]. Trastuzumab (TmAb) was successfully formulated as a high-concentration stable paste comprising more than 400 mg/mL TmAb concentration via XeriJect^®^ [66]. Regarding injectability, due to the shear-thinning properties, the paste formulations remained injectable. In a paste formulation containing immune globulin at 310 mg/mL with triacetin as the vehicle, the mean glide forces at a flow rate of 5.88 mL/min were 19 N via a 23 G needle, and 45 N via a 27 G needle [66]. Moreover, Xeris previously announced several research or license agreements using XeriJect^®^ with a number of pharmaceutical companies, including Amgen, Merck, and Regeneron [67]. To date, no clinical trials have yet been reported.

Although not yet being applied in developing high-concentration protein suspension, spray-freeze drying [68], electrostatic spray drying [69], as well as spray cooling (spray congealing) [70], have been explored for processing proteins and producing spherical protein particles. Spray-freeze drying involves atomizing a protein solution into a cryogenic liquid to form frozen droplets, followed by lyophilization to remove water from the system [68]. Spray-freeze drying produces spherical protein particles at low temperatures, favorably for thermally labile protein, although the particle size is typically larger than spray-dried particles [68]. However, the overall process time is substantially longer than that of spray drying alone. Electrostatic spray drying is similar to conventional spray drying but incorporates an electrostatic field, which can enhance water removal from droplets and thereby reduce the required drying temperature [69]. However, additional optimization of the applied charge is needed, and without such optimization, the yield may be much lower than that of conventional spray drying [69]. Spray cooling, or spray congealing, is essentially a solvent-free encapsulation process in which protein solids are either premixed with or dissolved in molten carriers, typically lipids at temperatures above the carrier melting point, and the mixture is then atomized into a cooling chamber maintained below the carrier melting point, where the mixture droplet solidifies into particles [70,71]. Although this method has been optimized to achieve high protein loading (90%) and suitable for proteins sensitive to moisture, the elevated pre-spraying temperature may still introduce thermal stress to the protein [70,71]. In addition, spray cooling typically requires protein solids as the starting material to enhance protein loading, which would necessitate a prior drying step for most therapeutic proteins.

Overall, given the scalability and capability of continuous manufacturing, spray drying remains a promising platform in producing high-concentration protein suspension injectables. Given that sufficient stabilizing excipients are required for spray drying, novel excipients such as MoNi, which can provide effective protection and particle surface coverage at low concentrations, are worth further development to enhance the clinical utility of such formulations.

### 2.4. Other Commercial Platforms

#### 2.4.1. Microglassification^TM^ by Lindy Biosciences

Microglassification^TM^ is a continuous, scalable, gentle, and rapid drying process at ambient temperature, leading to the formation of spherical dense solids [72]. It has been reported to successfully dehydrate biomolecules without structural damage, including enzymes, BSA, and mAbs [72,73,74,75]. Briefly, a micropipette is used to draw a desired volume of protein solution into the tip, which is then gently expelled into a non-aqueous vehicle such as decanol or pentanol to form a single microdroplet. The droplet remains attached to the pipette tip while water from the aqueous droplet diffuses into the organic phase, causing the droplet to shrink and ultimately solidify into a protein bead within seconds. The process can be scaled up to produce protein beads at a bulk scale using pentanol. The beads are collected via centrifugation to remove the supernatant (the organic phase), and placed under vacuum to remove any residual non-aqueous vehicle [72]. These protein beads turn out to be spherical, dense, and highly concentrated in protein.

In a previous publication on Microglassification^TM^ using BSA, the resulting microparticles were smooth and spherical, with most particles exhibiting diameters in the range of 10–20 µm [72]. The BSA concentration in the resulting microspheres reached as high as 1147 mg/mL, corresponding to only ~447 water molecules per BSA molecule—below the water amount required to form a monohydration layer around BSA [72]. This demonstrates the remarkable dehydration efficiency achieved by the Microglassification^TM^ process.

Moreover, the stabilizing effect of this dehydration method has also been demonstrated in different publications [72,75,76]. The Microglassified^TM^ BSA only had 2.7% irreversible aggregation [72]. The changes in the secondary structure were able to be reverted to the native condition upon rehydration [72]. In another study where the stability of a mAb molecule was compared upon Microglassification^TM^ vs. lyophilization, Microglassification^TM^ showed comparable stability profiles (monomer fractions, charge variants, and antigen binding) as lyophilization, except that subvisible particles were slightly higher [75]. Moreover, Hutcheson et al. employed Liquid-Observed Vapor Exchange Nuclear Magnetic Resonance (NMR) spectroscopy to analyze protein solids made out of different methods, including Microglassification^TM^, lyophilization, and vacuum drying [76]. The NMR data indicated that Microglassification^TM^ provided even better protein protection of protein native structure than lyophilization, as evidenced by a reduced number of locally unfolded protein residues following Microglassification^TM^ [76]. Together, these findings suggest that Microglassification^TM^ is a viable approach for producing stable biologic solids.

The ultra-high particle protein loading upon Microglassification^TM^ suggests its strong potential for developing high-concentration non-aqueous suspensions. However, to date, no published studies have reported the use of Microglassified^TM^ particles in any non-aqueous suspension formulations. Interestingly, in a recent publication, Zheng et al. have combined the protein–hydrogel particle approach (described in Section 2.1) with Microglassification^TM^ and successfully produced a high-concentration, stable, and injectable aqueous particle suspension [77]. This represents the first report of an aqueous antibody formulation achieving an ultra-high protein concentration of 360 mg/mL, a level comparable to that of the reported non-aqueous protein suspensions. Briefly, one inlet containing the antibody (amorphous IgG solids)-prepolymer mixture (dispersed phase) and two inlets containing pentanol (continuous phase) were combined at a microfluidic cross-junction [77]. Upon mixing, Microglassification^TM^ induced droplet dehydration, followed by further polymerization into hydrogel microparticles [77]. The hydrogel particle size was governed by the flow rate ratio of the continuous phase to the dispersed phase, with higher ratios producing smaller particles [77]. Particle diameters below 100 µm were achieved [77]. Injection force for the 360 mg/mL IgG-hydrogel suspension (particle loading 486 mg/mL) via a 25 G needle at 1.5 mL/min was below 20 N [77].

#### 2.4.2. Hypercon^TM^ by Elektrofi

Hypercon^TM^ is another proprietary method to formulate high-concentration protein microparticle for non-aqueous suspensions. Basically, droplets are generated from aqueous protein formulations and gently dehydrated to form high protein-loaded microparticle solids. To ensure a high protein-load particle production, a high protein concentration in the initial aqueous phase is preferred [78]. The collected microparticles are resuspended in the appropriate non-aqueous vehicles and concentrated to the desired concentrations.

In a recent publication, hIgG or rituximab microparticles were generated using this process [78]. These antibody particles were smooth and spherical with particle protein loadings of 64–78% (*w*/*w*) and residual moisture contents of 4% to 5% (*w*/*w*) [78]. The hIgG particles had a D_50_ of 13 µm [78]. These particles were suspended in a non-aqueous vehicle (not disclosed) to prepare suspensions [78]. The resulting hIgG microparticle suspension contained an antibody concentration of 500 mg/mL, with protein monomer fractions of approximately 93% [78]. The average injection force for the 500 mg/mL IgG microparticle suspension was below 20 N when delivered using a commercial prefilled syringe system fitted with a 27 G needle (flow rate not specified but above 6 mL/min) [78]. Furthermore, suspension formulations of hIgG or rituximab demonstrated comparable pharmacokinetics in rats and immunogenicity in mice to those of the corresponding aqueous formulations [78]. These data collectively demonstrate the potential of this platform technology for the development of high-dose and low-volume pharmaceutical injectables.

#### 2.4.3. Nanoform Platform

Nanoform also develops a proprietary platform to enable ultra-high-concentration biological suspension formulations above 400 mg/mL by producing spherical biomolecular particles with diameters as small as 50 nm from an aqueous phase, while maintaining protein stability [35]. These nanoparticles can be further dispersed in a non-aqueous vehicle to produce suspensions with shear-thinning properties to ensure acceptable injectability.

According to a reported case study on trastuzumab (TmAb) [79], TmAb particles had sizes ranging from 100 nm to 5 µm, and a D_50_ of 600–700 nm. These particles were subsequently suspended in benzyl benzoate, achieving a mAb concentration of 450 mg/mL (corresponding to a particle concentration of 650 mg/mL). The injection force with this formulation via a 27 G needle was below 10 N (flow rate not specified). However, to date, no published studies have reported the application of Nanoform particles in the development of these high-concentration protein suspensions.

Overall, these proprietary platform technologies have invested substantial effort in advancing high-concentration protein suspensions toward clinical application, often through partnerships with large pharmaceutical companies. Lindy Biosciences announced a licensing agreement and strategic collaboration on Microglassification^TM^ with Novartis in August 2024 [80]. In the same year, Lindy also announced the collaboration with Lifecore Biomedical, a contract development and manufacturing organization, to pursue the process development and commercial scale-up [80]. To date, no clinical trials have been reported on Microglassification^TM^ in development of high-concentration protein suspension injectables. With respect to Hypercon^TM^, Elektrofi was acquired by Halozyme in late 2025 [81]. Halozyme anticipated two programs using Hypercon^TM^ to enter clinical trial by the end of 2026 or earlier [81]. Nanoform has not reported any further progress beyond the case study described above regarding the high-concentration biological suspension platform [82].

### 2.5. Summary of Protein Particle Formation Techniques

In summary, a range of protein particle production methods has been established and evaluated for the development of high-dose, low-volume suspension injectables. Table 4 compares reported aqueous protein suspensions and non-aqueous suspensions with respect to particle production method, suspending vehicle, and protein concentration. Table 5 summarizes and compares these techniques. Because Elektrofi and Nanoform have not disclosed detailed information on their production processes, they are not included in Table 5. To more fully establish these particle production methods and improve their industrial translation, particularly for subcutaneous protein injectable products, key considerations include scalability, adaptability, compatibility with aseptic and continuous manufacturing, batch-to-batch reproducibility, and, where relevant, residual solvent control.

Although protein crystallization (discussed in Section 2.1) can produce dense and stable protein crystals, it is not yet readily predictable, scalable, or transferable across different protein drugs, particularly given the structural complexity and large size of therapeutic proteins such as mAbs. For preparing crystalline protein suspensions, the purity and batch-to-batch reproducibility of protein crystals should be carefully controlled. In addition, centrifugation is often required to formulate high-concentration protein suspensions in the current reports, which limits compatibility with continuous manufacturing. Recent efforts have explored the use of protein crystallization in downstream purification processes [83] as well as the development of continuous crystallization of proteins [84], which suggests the potential of protein crystallization for scalable and aseptic manufacturing. As these technologies continue to advance, crystalline protein suspensions may become increasingly attractive for concentrated protein suspension development.

Milling of protein lyophilizates (discussed in Section 2.2) may be more compatible with existing aseptic manufacturing because lyophilization is already a well-established technique for sterile parenteral protein products. However, the overall workflow is multistep and discontinuous, involving additional post-lyophilization unit operations such as milling and sieving, which may further increase batch-to-batch variability. Moreover, the irregular, flake-like morphology typically produced by milling can compromise injectability and reduce method adaptability across different proteins. Collectively, these factors may limit its readiness for broad industrial translation of high-concentration protein suspensions.

Spray drying (discussed in Section 2.3) is a one-step and readily scalable protein particle production technique that is also compatible with aseptic manufacturing. In addition, key particle attributes can be modulated through formulation composition and process parameters, highlighting the controllability of the spray drying process. Process controllability may support improved reproducibility of spray-dried particles. Moreover, spray-dried protein particles can exhibit desirable particle size distributions and spherical morphology, which are advantageous for subcutaneous injection. Taken together, spray drying appears to offer a more favorable balance of scalability, compatibility with aseptic and continuous manufacturing, and potential batch-to-batch reproducibility for high-concentration protein suspension development than protein crystallization or milling of protein lyophilizates. Its application by Xeris in the development of high-concentration protein suspension further supports the industrial translational potential of this approach relative to the other reported methods.

Microglassification^TM^ has demonstrated superior dehydration efficiency and solid protein stability in previous reports (discussed in Section 2.4.1). However, its broader industrial translation will require further demonstration of scalability and compatibility with aseptic and continuous manufacturing. In addition, the incorporation of organic solvents (e.g., decanol or pentanol) raises concerns regarding residual solvent control, which would need to be carefully addressed.

## 3. Impact of Particle Properties on High-Concentration Protein Suspensions

Regardless of particle production techniques, particle properties, including particle size distribution, particle morphology, and particle density, often correlate with suspension performance from different perspectives, including viscosity, injectability, and sedimentation.

Viscosity characterizes the resistance of a suspension to flow under applied stress [85]. It is a critical rheological property governed by the characteristics of the dispersed particles and the continuous phase (the suspension vehicle), as well as by particle–particle, particle–vehicle, and vehicle–vehicle interactions [26,86,87]. Viscosity also directly impacts injectability and the sedimentation behavior [64,88].

Injectability can be described as the ease with which a suspension can be delivered through a needle and syringe or injection device, characterized by the force required to initiate and maintain flow of the formulation during injection [89]. A successfully developed suspension product for subcutaneous delivery can be fully administered with acceptable dose accuracy into the patients’ subcutaneous tissue using forces that are tolerable to the healthcare professionals or patients, typically up to 38–50 N, based on human factors studies evaluating syringe injection difficulty [55].

Sedimentation in a suspension refers to settling dispersed solid particles in the continuous phase under gravity, which may occur during storage or clinical dosing. A slower sedimentation rate is generally preferred to maintain physical stability and dose uniformity; however, if sedimentation does occur, the resulting sediment may be compact and difficult to resuspend [90].

This section provides an overview of how particle properties influence the aforementioned suspension formulation behaviors. Understanding the relationships between particle properties and suspension behaviors can guide the rational design of formulations with desirable attributes (Table 6).

**Table 6:** Correlations between particle properties and suspension properties.

	Suspension Performance	Viscosity	Injectability	Sedimentation
Particle Properties				
Particle size		Larger particles may reduce suspension viscosity by reducing interparticle interactions (increasing interparticle distance and reducing particle surface area).	Larger particles increase the risk of needle clogging. If no clogging occurs, larger particles may decrease injection force.	Larger particles increase the risk of faster settling rates (Equation (1)).
Particle morphology (spherical vs. non-spherical)		Spherical particles present lower viscosity by increasing particle packing efficiency (Equation (1)).	Spherical particles reduce the risk of particle jamming and needle clogging. Spherical particles may reduce injection force.	Spherical particles have less settling drag than the non-spherical ones (Figure 1).
Particle density		Not directly influence viscosity.	An increase in particle density may reduce the risk of particle jamming and needle clogging by increasing particle inertia.	The larger the difference in densities between the particle and the vehicle, the larger the settling rate is (Equation (1)).

**Figure 1:**
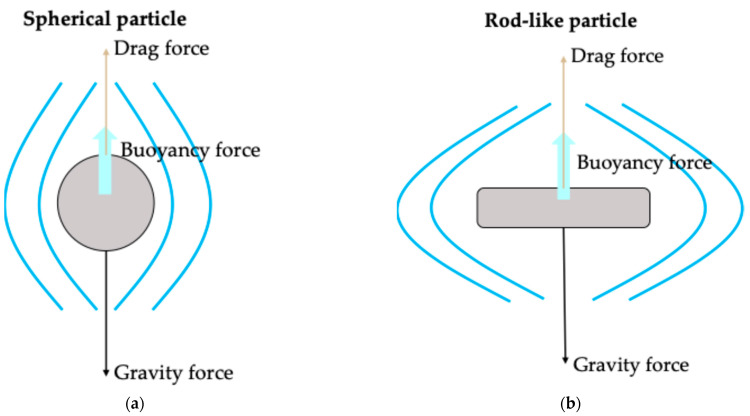
Illustration of single spherical (**a**) vs. rod-like (**b**) particles settling in an unbounded Newtonian fluid. The downward black arrow indicates the gravitational force (F_g_) due to the particle weight. The upward brown arrow represents the drag force (F_d_) caused by fluid resistance, while the upward blue arrow represents the buoyant force (F_b_) exerted by the surrounding fluid. The net settling force (F_s_) can be expressed as: F_s_ = F_g_ − (F_d_ + F_b_).

### 3.1. Impact of Particle Size and Particle Size Distribution

Protein particles produced from the methods described in Section 2 typically exhibit a range of particle sizes and a corresponding size distribution. Regarding the impact of particle size on the suspensions, Liu et al. recently published a review article on long-acting injectables [55]. In terms of viscosity, 20 cP is a general upper limit for subcutaneous solution injectables [91,92]. As for the concentrated suspensions, there are several equations to predict the viscosity [93]. Among these equations, there is a widely used model proposed by Krieger and Dougherty for describing viscosity in suspensions of rigid spherical particles [94]:

(1) $$\frac{\eta }{\eta_{0}}={\left(1-\frac{\varphi }{\varphi_{max}}\right)}^{-\left[\eta \right]\varphi_{max}}$$

where η is the system viscosity; $\eta_{0}$ is the vehicle viscosity; $\left[\eta \right]$ is intrinsic viscosity, depending on particle morphology (the lowest value is 2.5 for rigid spheres); φ is the particle volume fraction; and $\varphi_{max}$ is the maximum particle packing fraction. According to this equation, at the same particle volume fraction, a higher maximum particle packing efficiency decreases the system’s relative viscosity, as an increase in $\varphi_{max}$ indicates that particles can be packed to a higher extent before the suspension becomes jammed. For a polydisperse particle suspension, an increase in the width of particle size distribution increases particle size variability (small particles can occupy the space between the large ones), leading to a better packing efficiency and a lower viscosity [95,96,97]. Regarding particle size diameter, although it is not explicitly included in Equation (1), several studies have suggested that particle size diameter can also influence the suspension viscosity, with a reduction in the particle size leading to increased suspension viscosity [28,86,98,99]. At the same volume fraction, an increase in particle size increases the interparticle distance and thereby reduces the interparticle interactions, such as collision frequency [64]. Reducing interparticle interactions can decrease viscosity. Overall, viscosity is determined by the combined influence of particle properties and vehicle properties. More investigations are still needed to better model the viscosity in different concentrated suspensions.

With respect to suspension injectability, high injection force and particle-induced needle clogging are the two primary contributors to product failure. Regarding the injection force for subcutaneous administration, the maximum acceptable range is between 38 N and 50 N based on human factor studies [55]. Particle size and particle size distribution can influence suspension injectability by affecting viscosity, as a higher viscosity can increase the injection force [64]. In a study on a microsphere suspension system, Zhao et al. applied levonorgestrel particles of different mean particle sizes (50 µm, 100 µm, 150 µm, 200 µm, and 250 µm), and further dispersed the particles in sodium carboxymethyl cellulose (NaCMC) solution at 120 mg/mL [89]. During the injectability test using a 23 G needle at 150 mL/min, it was found that particle size did not affect the injection force within a certain range, and led to a sudden increase after exceeding 150 µm [89]. Overall, large particle size tends to increase injection force and lead to injection failures. In addition, larger particles increase the risk of needle clogging more than the smaller ones [89,100]. Particle sizes below one-third of the needle inner diameter are considered less likely to induce needle clogging [101]. Kowsari et al. evaluated the injectability of suspensions containing glass spheres with diameters of 12, 25, and 35 µm, respectively, at a fixed concentration of 350 mg/mL [100]. It was found that the incidence of needle clogging during injection through a 27 G needle increased with particle size [100]. It should be noted that the levonorgestrel particles and the glass spheres studied in the two referenced works cannot fully represent the physiochemical properties of protein particles [89,100]. However, because these particles share similarities in morphology and particle size range with those reported for high-concentration protein suspensions, the findings may still provide valuable insights for guiding further optimization in future studies, particularly given the limited number of published studies currently available in this area.

In addition, particle size also affects the suspension sedimentation behavior. In general, suspensions containing particles with a diameter less than 1 µm are considered colloidal systems, and they do not sediment over time due to Brownian motion [102]. Stokes’ Law (Equation (2)) describes the settling rate of smooth solid spheres in a unbounded Newtonian liquid, assuming no particle–particle interactions [88]:

(2) $$v=\frac{d^{2}\left(\rho_{p}-\rho_{f}\right)g}{18\eta }$$

where v is the settling rate; d is the particle diameter; $\rho_{p}$ is the particle density; $\rho_{f}$ is the vehicle density; η is the vehicle’s Newtonian viscosity. Reducing particle size can slow down the sedimentation rate. However, in systems like high-concentration particle suspensions, which behave more as a non-Newtonian system, Stokes’ law alone is insufficient to predict or explain sedimentation behavior [90]. Sedimentation is also governed by the particle concentration, particle morphology, particle surface charge, and other factors [103]. Furthermore, in a study on crystalline mAb suspensions, it was found that the 50 mg/mL mAb suspension with a homogeneous monomodal particle size distribution (39 µm) sedimented faster and formed a more uniform sediment than the one with a heterogeneous bimodal particle size distribution (13 µm and 102 µm) [40]. This could be attributed to the difference in viscosity arising from the different particle size distributions. Overall, larger particles tend to increase the risk of sedimentation, and particle size can also influence the sedimentation of suspensions by affecting viscosity.

### 3.2. Impact of Particle Morphology

Particle morphology influences suspension viscosity. At a given volume fraction, particle morphology affects the maximum packing fraction ($\varphi_{max}$; Equation (1)) and, consequently, viscosity. For monodisperse spherical particles, the theoretical maximum packing fraction is approximately 0.74 for ordered packing, 0.64 for random close packing, and 0.56 for random loose packing [104,105]. Increasing particle anisometry, corresponding to greater deviation from spherical shape, reduces the maximum packing fraction and consequently increases suspension viscosity [90,104]. In addition, increasing particle anisometry enhances particle–particle interactions and further increases viscosity, as non-spherical particles occupy a larger effective volume during rotation than spherical particles, thereby increasing the likelihood of contact with neighboring particles [104]. Collectively, at the same volume fraction, suspensions composed of spherical particles exhibit lower viscosity than those containing non-spherical (e.g., prolate or oblate) particles.

With respect to suspension injectability, spherical particles have also been reported to reduce the risk of particle jamming and needle clogging more than non-spherical particles, like plate-shaped or fiber-shaped particles [28,100]. During the injection of concentrated suspension formulation, particles stack and bridge to one another at the needle entrance, resulting in particle jamming and needle clogging [100]. Needle clogging is typically characterized by an abrupt peak in the injection force profile [100]. Interestingly, fiber-shaped particles were reported to stack within the needle without necessarily causing complete clogging when the particle solid volume fraction was reduced from 10% to 2.5% [100]. Instead, they created a filtering effect that only allowed the vehicle’s liquid to pass through [100]. Nevertheless, particle jamming will still lead to dose inaccuracy for the pharmaceutical injectables.

In terms of sedimentation, at the same volume fraction and with comparable particle sizes, increasing particle anisometry can lead to the possibility of higher settling drag and thereby reduce settling rates [106]. For example, when rod-like particles settle in a flat orientation relative to the direction of motion, the drag force increases, further slowing sedimentation (Figure 1).

Overall, spherical particles outperform non-spherical particles in developing a low-viscosity and injectable suspension. They may present faster sedimentation than non-spherical ones under certain conditions.

### 3.3. Impact of Particle Density

Particle density can influence the effective particle volume fraction and thereby indirectly affect suspension viscosity, as denser particles reduce void space and increase packing density.

With respect to injectability, Kowsari et al. utilized hollow and solid glass spheres of identical particle size (13 µm) to vary the particle density and evaluate the injectability under the same condition [100]. It was observed that solid glass spheres eliminated clogging risks [100]. This injectability improvement could be attributed to the higher particle density, which increased particle inertia and decreased the tendency of particles to follow fluid streamlines [100]. As a result, fewer particles accumulated at the needle-syringe constriction, thereby reducing the risk of needle clogging [100].

In terms of sedimentation, according to Stokes’ Law (Equation (2)), an increase in particle density increases the sedimentation rate. For example, the spray-dried lysozyme powder with a density of 1.3 g/cm^3^ exhibited flotation in perfluorodecalin with a density of 1.9 g/cm^3^, and sedimented in other vehicles (e.g., perfluorobutylpentan [F4H5]) with a density lower than the particle at 70 mg/mL protein concentration upon one-hour room-temperature (RT) storage [28]. All the suspensions were resuspendable [28]. However, such settling (flotation) due to density difference was reduced when protein concentration was above 140 mg/mL [28]. This could be attributed to the higher particle concentration, which increases the suspension viscosity and reduces sedimentation.

### 3.4. Interplay Between Particle Properties and Suspension Performance Attributes

In summary, particle properties are closely interconnected with suspension performance, including viscosity, injectability, and sedimentation. Among these performance attributes, viscosity plays a central role because it reflects the overall rheological behavior of the suspension and strongly influences both injectability and sedimentation.

The key motivation for developing protein suspensions is to address the limitations of high-concentration protein solutions, including high viscosity and protein instability, while establishing a platform approach that can be readily transferred and applied across different biologic modalities. Although the studies discussed in Section 2 have demonstrated improvement in viscosity and injectability using concentrated protein suspensions, further mechanistic investigation is warranted to more fully establish these formulations as a viable drug product platform.

Suspensions may behave as non-Newtonian fluids, indicating that their viscosity is shear-rate dependent. Therefore, improved understanding of suspension viscosity will require the development of advanced viscosity models tailored to these systems, since most existing models are based on hard-sphere assumptions [93]. However, the protein particles discussed in Section 2 are not hard, rigid spheres. Moreover, these particles are not always perfectly spherical; for example, spray-dried particles may exhibit surface features such as dimpling [27]. In addition, protein particle surfaces can be heterogeneous and may exhibit complex interparticle interactions in concentrated suspensions. Incorporating these particle-related considerations, including particle softness, particle surface composition, particle surface features, and interparticle interactions, into future viscosity predictive work will provide meaningful insight into the development of concentrated protein suspensions and facilitate clinical translation.

Another important consideration is the risk of particle jamming and needle clogging during suspension injection, which may occur despite low suspension viscosity. Typically, the shear rates during subcutaneous injection can be as high as 10^5^ s^−1^ [65]. During injection, the suspension is forced through the constricted geometry of the syringe–needle system. System geometry, interparticle interactions, particle–wall interactions, and the sudden increase in shear rate may promote particle structure deformation, particle bridging, thereby increasing the likelihood of particle jamming and needle clogging [100]. Therefore, particle properties again play a critical role in controlling the risk of needle clogging.

With respect to sedimentation, although minimizing sedimentation is generally preferred, additional consideration of resuspendability would add value to future studies, particularly for protein products intended to be manufactured and stored as suspensions rather than in powder-in-vial form. With regard to resuspendability testing, the United States Pharmacopeia (USP) <1003> may serve as useful guidance. Long-term storage may increase the likelihood of sedimentation, and poor resuspendability may compromise product quality and lead to dose inaccuracy. In general, loosely packed sediment is more readily redispersed. However, the effects of particle properties on resuspendability in concentrated protein suspensions remain poorly understood and require further investigation. Although not fully representative of protein suspensions, one study on the resuspendability of aluminum-adjuvanted vaccine formulations hypothesized that resuspendability was associated with particle size distribution, with higher fine-to-large particle size ratios leading to increased packing efficiency, as fine particles can fill the void spaces between larger particles, and ultimately reduced resuspendability [107].

Overall, particle properties provide a means to tune suspension performance. It should be noted that although particle density can influence suspension performance, modifying it during drug product development is often constrained by formulation composition and particle production methods, and cannot be readily adjusted to achieve meaningful performance improvements. In contrast, particle size and particle morphology are more readily tunable parameters for improving suspension performance. Overall, spherical particles smaller than 20 µm are considered desirable for developing high-concentration protein suspensions for subcutaneous injections.

## 4. Critical Considerations for the Successful Development of High-Concentration Protein Suspensions

In summary, the successful development of high-concentration protein suspensions depends critically on the appropriate selection of particle production methods. This decision is multifaceted and depends on (1) a strong understanding of particle formation processes, (2) a clear understanding of the relationship between particle properties and suspension performance, and (3) the readiness of the technique for manufacturability and industrial translation.

Once the protein particle production method has been selected, either aqueous or non-aqueous vehicles may be used to prepare protein suspensions depending on particle properties. Compared with aqueous vehicles, however, non-aqueous vehicles may raise additional regulatory concerns, particularly with respect to injection-site pain, local irritation, inflammation, and systemic toxicity. Ideal non-aqueous vehicles for subcutaneous injection should be pharmacologically inert, safe for patients, sterilizable, compatible with the protein drug, and sufficiently low in viscosity. In addition, prior use of a non-aqueous vehicle in approved parenteral products may help facilitate regulatory translation, whereas other vehicles may require more extensive safety and tolerability justification, potentially prolonging development timelines. A review by Marschall et al. summarizes potential non-aqueous vehicles for use in protein suspensions [45].

Following selection of the protein particle production method and suspension vehicle, consideration should also be given to the final product presentation, such as powder-in-vial products or suspensions. For powder-in-vial products, powder filling becomes a critical manufacturing step. In this context, free-flowing and non-cohesive powders are preferred to facilitate accurate filling and minimize product loss. Before administration, the ability of the powder to redisperse uniformly in the vehicle is also critical to product quality. In practice, manual shaking or inversion may be the most feasible methods for redispersion. In addition, syringeability and injectability directly affect the usability of the final product.

For suspensions, scalable and manufacturing-compatible mixing processes are preferred. Two main approaches may be considered: (a) filling the powder and vehicle separately into storage vials or injection devices, followed by homogenization; or (b) homogenizing the powder and vehicle first, followed by filling the suspension into vials or injection devices. Additionally, long-term stability tests on sedimentation, injectability, and syringeability should be evaluated. Therefore, different product presentations introduce distinct formulation and manufacturing considerations. Overall, ready-to-use suspensions in injection devices may represent a promising strategy for the development of self-administration products. Future studies should further evaluate the compatibility of suspension formulations with currently available injection devices.

Furthermore, for the manufacturing of any injectable product, sterility must be carefully controlled to minimize the risk of microbial contamination [55]. A terminal sterilization procedure, such as heat sterilization or gamma radiation, may be required for suspension products, although such processes may alter particle properties and suspension performance [55,108,109]. These effects warrant further investigation. In addition, aqueous protein suspensions may be more susceptible to microbial contamination during storage than non-aqueous protein suspensions. Therefore, appropriate sterility testing should be incorporated to monitor the manufacturing process, support product release, and evaluate long-term stability in order to establish suitable storage conditions [55].

With respect to defining appropriate critical quality attributes for the development of protein suspensions, further studies are still needed. As discussed in this review, particle size and morphology may be critical for injectability. Suspension viscosity may also be a critical attribute because it plays a central role in injectability, syringeability, sedimentation, and manufacturability. Moreover, injectability, syringeability, and resuspendability, when applicable, also constitute key quality attributes. Therefore, robust and advanced analytical characterization of suspension formulations is needed to achieve a mechanistic understanding of these systems and to define relevant quality attributes.

Protein stability and biological activity should be maintained throughout suspension formulation development, including process stability during particle production and suspension preparation, as well as storage stability during shipping, storage, and administration. Changes in protein stability or activity may raise concerns regarding product safety and efficacy. For example, protein aggregates may increase immunogenicity risk or alter protein activity [110,111].

Moreover, it is critical to demonstrate comparability between protein suspensions and their corresponding solution formulations with respect to bioavailability, pharmacokinetic profiles, and immunogenicity. Ideally, protein suspensions should readily dissolve and be absorbed following subcutaneous injection. In vitro dissolution or release should ideally reach 100% within an appropriate time frame, and relevant testing methods may be informed by USP <711> and <1001>. In addition, the presence of protein particles and non-aqueous solvents may induce local inflammatory response, underscoring the importance of tolerability assessment during formulation development. To date, crystalline protein suspension [23,42], spray-dried protein suspension [62], as well as Hypercon^TM^ protein suspension [78] have been reported to exhibit comparable bioavailability and no increased inflammation compared with corresponding solution in animal studies. These findings suggest that the protein particles are readily dissolved upon subcutaneous injection and absorbed into the systemic circulation without causing local inflammation.

Overall, the successful development of protein suspension injectable products requires integrated consideration of protein particle production, suspension vehicle selection, final product presentation, manufacturability, and maintenance of drug product safety, efficacy, and quality throughout the development process. Continued advances in industrial translation and relevant regulatory science will be important for supporting the progress of protein suspensions into clinical trials and commercialization.

## 5. Conclusions and Future Directions

From a theoretical standpoint, the maximum achievable concentration of mAb in aqueous solution is fundamentally constrained by molecular packing limits [11]. Assuming the mAb molecules are closely packed as identical spheres in a face-centered cubic lattice, the theoretical upper bound is approximately 440 mg/mL [11]. In practice, however, the maximum attainable concentration of mAb solutions is considerably lower than 400 mg/mL due to limitations imposed by solubility, viscosity, and protein stability. These intrinsic constraints underscore the challenges of further increasing doses using the solution-based formulations.

Protein suspensions offer a viable approach to overcome these limitations by enabling drug concentration beyond the solubility limits of solution-based systems, with reported protein concentrations reaching up to 500 mg/mL [62,78]. As interest in low-volume, high-dose subcutaneous drug products continues to grow, protein suspensions are increasingly recognized as a promising platform for next-generation injectable biologics. Accordingly, further expansion of research and development efforts in this area is anticipated in the coming years.

For future directions, given the total number of available reports so far, several considerations and critical challenges must be addressed to enable the successful translation of high-concentration protein suspensions into commercialized drug products.

- **Advanced protein particle design**: Although current understanding has started to define preferred particle properties, particularly in terms of size and morphology, for improved suspension performance, further studies are needed to elucidate the effects of particle properties on protein stability, biological efficacy, pharmacokinetic behavior, and immunogenicity.
- **Balancing injectability and protein stability**: In ultra-high-concentration suspension formulations remains a key challenge, particularly with respect to excipient selection and optimization of excipient-to-protein ratios. Higher particle drug loading is desirable for the development of high-dose therapeutics. Furthermore, improving formulation stability to achieve long-term room-temperature storage would substantially enhance clinical utility and further facilitate self-administration.
- **Suspension vehicle selection and regulatory considerations**: The majority of protein suspensions reported to date rely on non-aqueous vehicles. Identifying vehicles that preserve protein integrity, minimize injection-site pain, and ensure patient safety is critical. At the same time, the need for additional regulatory justification for non-aqueous excipients may extend development timelines and should be considered early in formulation strategy design. Importantly, inclusion of a vehicle in a formulation does not imply equivalent regulatory readiness; prior parenteral use and local tolerability remain key constraints.
- **Establishing a mechanistic understanding and leveraging advanced analytical characterization**: A deeper mechanistic understanding of how particle attributes correlate with the overall suspension performance is needed. While relevant concepts have been developed in other fields, their applicability and feasibility to protein suspensions remain limited and insufficiently validated. Moreover, the characterization of highly concentrated suspensions containing fine particles presents unique analytical challenges that require further methodological development and leveraging advanced analytical techniques.
- **Manufacturability and scalability**: Robust, scalable mixing and homogenization strategies are essential to ensure formulation reproducibility and manufacturability while minimizing material loss. Many laboratory-scale studies reviewed herein rely on solely vortex mixing, which is not suitable for large-scale production, underscoring the need for the development of industrially relevant processing approaches. In addition, sterilization is required for injectables, but suspensions face challenges with terminal sterilization or sterile filtration as they may negatively affect the quality attributes, stability, and potency of the suspension drug product or lead to filter clogging. Therefore, high-concentration protein suspensions demand specialized manufacturing facilities, equipment and technology as well as more investment in process development and characterization.
- **Device compatibility and patient use**: Compatibility with existing injection devices, including prefilled syringes and autoinjectors, is critical for clinical translation. While protein suspensions may be developed as either powder-in-vial or ready-to-use formulations, ready-to-use formulations are generally preferred for self-administration due to reduced preparation steps and lower risk of dosing errors.
- **Biopharmaceutical and pharmacokinetic understanding:** In contrast to the rapid diffusion and uptake into blood or lymph of the dissolved molecules in solutions, upon injection, the solid protein particles in suspension must first undergo dissolution prior to diffusion and absorption. The dissolution step may be rate-limiting, resulting in slower and longer absorption. Particle properties again play a role in governing these kinetics. Consequently, the suspension pharmacokinetics is less predictable than solutions and warrants more investigation.
- **Product quality control:** Although crystalline insulin suspension products for subcutaneous injection are commercially available, they are formulated at relatively low protein concentrations (100 units/mL insulin for HUMULIN^®^ N [approximately 3 mg/mL pure crystalline insulin]) [112], and there are no marketed high-concentration protein suspension drug products with protein concentrations exceeding 100 mg/mL. Hence, limited knowledge and experience are available on the relevant product quality control. The pharmaceutical industry and regulatory authorities should collaborate to determine the proper critical quality attributes to be characterized and sufficient analytical release and stability data to be acquired and reviewed in order to ensure adequate surveillance on product quality and safety for the patients [113].

In summary, high-concentration protein suspension formulations represent a compelling and evolving approach to enable patient-centric, high-dose subcutaneous delivery of biologics. Continued advances in formulation science, analytical methodologies, processing technologies, device integration, pharmacokinetic understanding, and regulatory guidance will be required to fully realize the potential of this platform and support its broader adoption in commercial drug products.

## Figures and Tables

**Table 1:** Summary and comparison of publications on high-concentration protein–hydrogel particle aqueous suspensions.

	Study 1	Study 2	Study 3
Protein precipitation to generate crystalline or amorphous proteins	Monoclonal antibody (mAb) crystals were batch crystallized and grown in a buffer containing polyethylene glycol (PEG).	MAb crystals were batch crystallized and grown in a buffer containing PEG.	Pembrolizumab and human IgG were each precipitated in a buffer containing PEG to form amorphous antibody solids.
Prepolymer preparation	Poly(ethylene glycol) diacrylate (PEGDA) and Darocur 1173 * were added to the concentrated mAb crystalline suspensions to prepare the prepolymer mixture.	Sodium alginate (NaALG) prepared in the PEG buffer was added to the mAb crystal suspension and later centrifuged until reaching the desired concentration.	NaALG was added to the concentrated amorphous solid proteins to prepare the prepolymer mixture.
Protein–hydrogel microsphere formation process	Prepolymer droplets were formed and mixed with mineral oil in a microfluidic cross-junction. The droplets were polymerized under exposure to UV, leading to the formation of mAb crystal-hydrogel microspheres. Particles were collected and washed with a fresh PEG buffer ((4-(2-hydroxyethyl)-1-piperazineethanesulfonic acid) [HEPES] buffer containing PEG) multiple times. The resulting hydrogel suspension was centrifuged to the desired concentration.	Centrifugal extrusion was applied to form the microspheres. The prepolymer mixture was loaded into a syringe connected to a blunt-tip dispenser and positioned above a centrifugal tube containing the cross-linking Ca^2+^ solution. Upon centrifugation, the mixture was extruded via the dispenser to form antibody–hydrogel particles in the cross-linking bath. The cross-linking solution was subsequently replaced with the PEG buffer (HEPES buffer containing PEG) to resuspend the particles.	Same as Study 2. Centrifugal extrusion was used to produce protein–hydrogel particles.
Protein–hydrogel particle properties	The resulting particle size diameter was dictated by the flow rate in the microfluidic mixer, ranging from 50 to 140 µm. The particles were spherical.	The resulting particle size diameter could be reduced by an increase in centrifugal forces, where the smallest was still above 100 µm. The particle morphology was affected by the distance between the dispenser and the crosslinking bath.	The particles were spherical and opaque with average diameters of around 220 µm, maintaining stable particle morphology for over 15 months at 4 °C.
Protein–hydrogel suspension properties	The suspensions were shear-thinning. The viscosity of a 300 mg/mL mAb-hydrogel particle suspension was approximately 35 centipoise (cP) at a shear rate of 4000 s^−1^, and it was manually injectable via a 26-gauge (26 G) needle.	The mAb-hydrogel suspension was injected up to 200 mg/mL mAb via a 27 G needle, while no injection profiles were reported.	The IgG-hydrogel suspension up to 250 mg/mL IgG was injectable via a 27 G needle, while no injection forces were reported. From 200 mg/mL, the suspension showed a paste-like texture.
Protein stability and binding activity	More than 93% mAb released from hydrogel particles remained monomeric. The mAb-loaded hydrogel particle preparation did not negatively affect binding efficacy.	No significant change in monomer fraction, binding activity, charge variant, or chemical modification was observed after protein–hydrogel particle production. Moreover, the particles did not induce cytotoxicity or immunogenicity in in vitro assays.	The mAb-hydrogel suspension at 200 mg/mL showed no change in monomer fraction after storage at 4 °C for 15 months. The antibody-loaded (both IgG and mAb) hydrogel particles did not negatively affect binding activity. Moreover, IgG hydrogel suspensions (200 mg/mL) maintained binding activity after storage at 25 °C for 100 days. The mAb-hydrogel suspension (200 mg/mL) after 15 months at 4 °C did not show significant reduction in binding activity.
In vitro release from hydrogel particles	In vitro release results (medium: phosphate-buffered saline; temperature not reported) suggested that while mAb crystal could fully be dissolved in the particles, and mAb slowly released from particles for up to 4 days after a burst release. At 200 mg/mL and 300 mg/mL, the mAb was not completely released from the hydrogel particles (~80%).	In vitro release study suggested that the crystalline mAb could be fully dissolved and completely released from the hydrogel particles within 60 min (dissolution medium: simulated body fluid; temperature 37 °C).	In vitro release study suggested both amorphous antibodies could be completely released from the hydrogel particles within 20 min (dissolution medium: simulated body fluid; temperature 37 °C).
In vivo evaluation	Not reported.	The mAb-hydrogel particle suspension upon subcutaneous injection showed comparable pharmacokinetic profiles as crystalline mAb formulation, demonstrating comparable bioavailability in rats.	Not reported.
Reference	[41]	[42]	[46]

* Darocur 1173 is a photoinitiator.

**Table 2:** Comparison of two milled lyophilized protein suspensions published by the same group.

	Study 1	Study 2
Particle production method	Lyophilized protein solid was cryomilled and then sieved through a 40 µm mesh for both studies.	
Protein particle composition	mAb-sucrose (7:3; *w*/*w*)	lysozyme–trehalose (7:3; *w*/*w*)
Particle size distribution	D_50_ = ~8 µm	D_50_ = 8.1 ± 0.7 µm
Particle morphology	Particles exhibited irregular, flake-like morphologies under SEM.	
Protein concentration in the suspension	150 mg/mL	210 mg/mL
Suspension vehicle and suspending method	Particles were mixed and homogenized with perfluorohexyloctane (F6H8) in the ultrasound bath.	
Viscosity	Below 10 cP at a shear rate of 5000 s^−1^	13.9 ± 0.8 cP at a shear rate of 5000 s^−1^
Injectability	Glide force below 20 N via a 25 G needle at a flow rate of 6 mL/min	Not injectable through a 26 G needle at a flow rate of 6 mL/min
Reference	[25]	[28]

**Table 4:** Comparison of aqueous protein suspension and non-aqueous protein suspension from the reported studies.

	Particle Production Method	Suspending Vehicle	Highest Protein Concentration Reported	Reference
Aqueous protein suspension	Protein crystallization	Phosphate buffer containing PEG and ethanol, HEPES buffer containing PEG	140 to 200 mg/mL	[23,40]
	Proteins (crystalline, amorphous, or Microglassified^TM^) are encapsulated into hydrogel particles	HEPES buffer containing PEG	200 to 360 mg/mL	[41,42,46,77]
Non-aqueous protein suspension	Protein precipitation to produce amorphous protein	Benzyl benzoate, ethyl lactate, tetrahydrofuran, toluene, acetonitrile, isopropanol, N-methylpyrrolidone, methyl ethyl ketone, decane, ethanol, methanol, PEG 200, propylene glycol, 1,4-butanediol.	260 mg/mL	[43]
	Milling of protein lyophilizates	F6H8, benzyl benzoate, toluene	150 to 400 mg/mL	[49]
	Spray drying	Benzyl benzoate Miglyol^®^ 840, ethyl lactate, triacetin, F4H5, F6H8, perfluorodecalin, ethyl oleate, Miglyol^®^ 812 *, sesame oil *	200 to 520 mg/mL	[26,27,28,62,66]
	Hypercon^TM^	Not disclosed	500 mg/mL	[78]
	Nanoform platform	Benzyl benzoate	450 mg/mL	[79]

* Miglyol^®^ 812 and sesame oil have viscosity of 23 cP and 51 cP, respectively. Suspension prepared in these two vehicles had high viscosity and high injection force.

**Table 5:** Comparison of different particle formation methods for the development of high-concentration biologic suspensions.

	Pros	Cons
Crystallization	Crystalline form of biomolecules is stable High-concentration aqueous suspensions are reported	Scalability Uniform and consistent crystallization of biomolecules like mAbs is challenging Protein crystallization is mostly unpredictable Not a continuous process (centrifugation is often applied to concentrate the formulation)
Milling of lyophilizates	Lyophilization is a well-established standalone drying technique with relatively fewer regulatory concerns	Not a continuous process Long production time Milling introduces additional mechanical stress, and a standardized milling procedure is preferred An additional sieving step may be required to ensure good injectability Particles are flake-like and irregular, potentially causing problems in injectability Non-aqueous vehicle is currently the primary option, which raises more regulatory concerns
Spray drying	A continuous process Rapid drying Scalable Aseptic operation available The stability of spray-dried biologic solids has been widely demonstrated Can generate small round particles suitable for subcutaneous injection Particle properties can be tailored	Thermal, shear, and interfacial stresses Adequate amounts of stabilizing excipients may compromise the drug loading in the particle and the resulting suspension Concerns regarding non-aqueous vehicles
Microglassification^TM^	Ambient temperature processing Rapid drying Superior dehydration and ultra-high particle drug loading Can generate small round particles suitable for subcutaneous injection	Concerns regarding non-aqueous vehicles Scalability needs to be validated

## Data Availability

No new data were created or analyzed in this study.

## Abbreviations

The following abbreviations are used in this manuscript:

BSA	Bovine serum albumin
cP	Centipoise
cryomilling	Cryogenic milling
FDA	The United States Food and Drug Administration
F4H5	Perfluorobutylpentane
F6H8	Perfluorohexyloctane
G	Gauge
HEPES	4-(2-hydroxyethyl)-1-piperazineethanesulfonic acid
IgG	Immunoglobulin G
mAb	Monoclonal antibody
MoNi	Poly(acryloylmorpholine-co-N-isopropylacrylamide)
NaALG	Sodium alginate
NaCMC	Sodium carboxymethyl cellulose
PEG	Polyethylene glycol
PEGDA	Poly(ethylene glycol) diacrylate
RT	Room temperature
TmAb	Trastuzumab
USP	The United States Pharmacopeia
